# Interprofessional Learning – Development and Implementation of Joint Medical Emergency Team Trainings for Medical and Nursing Students at Universitätsmedizin Greifswald

**DOI:** 10.3205/zma001031

**Published:** 2016-04-29

**Authors:** Maud Partecke, Claudius Balzer, Ingmar Finkenzeller, Christiane Reppenhagen, Ulrike Hess, Klaus Hahnenkamp, Konrad Meissner

**Affiliations:** 1 Universitätsmedizin Greifswald, Körperschaft des öffentlichen Rechts, Klinik für Anästhesiologie, Greifswald, Germany; 2 Berufliche Schule an der Universitätsmedizin Greifswald, Körperschaft des öffentlichen Rechts, Greifswald, Germany; 3 Universitätsmedizin Greifswald, Körperschaft des öffentlichen Rechts, Geschäftsbereich Pflege, Praxisanleitung, Greifswald, Germany

**Keywords:** Medical education, emergency medicine, patient simulation, interprofessional relations

## Abstract

**Introduction:** Interprofessional collaboration is of great importance in clinical practice, particularly in the field of emergency medicine. The professions involved in providing emergency care must work hand in hand, and tasks and routines must be coordinated effectively. However, medical and nursing students have only few opportunities to experience interprofessional cooperation during their formal training. Addressing this situation, the Department of Anesthesiology and the Vocational School of Greifswald University Medical School initiated a project to increase patient safety by integrating interprofessional human factor training into the curriculum of both health professions. This manuscript addresses how an interprofessional course module focusing on clinical emergency medicine can be taught with an emphasis on competency and problem-solving. In addition, it was important to identify suitable instruments for systematic quality development and assurance of this teaching and learning format.

**Project description: **The aim of the project, which took place from October 2013 to September 2015, was the development, implementation and evaluation of a simulation-based, interprofessional course module on clinical emergency medicine. Target groups were medical and nursing students. Modern pedagogical models and methods were applied to the design and teaching of the course content. The project was carried out in separate phases: definition, planning, practical implementation, evaluation and documentation. The project was accompanied by systematic quality development. Established guidelines for quality-centered school development were applied to quality development, assurance and evaluation.

**Results: **Over two years, a 16 credit-hour course module was developed and then taught and evaluated during the 2014 and 2015 summer semesters. A total of 120 medical students and 120 nursing students participated in the course module. Eighteen teachers from medicine and nursing were trained as instructors and assisted by 12 student tutors. Regular evaluations focused on different aspects of the project, using instruments for empirical educational research. Excellent ratings given to the course by the attendees indicate a high degree of satisfaction in both participating professions regarding course design and content, as well as the quality of teaching.

**Discussion: **In a position paper, the GMA committee on Interprofessional Education in Health Professions issued recommendations for interprofessional education. The recommendations given for teaching and quality assurance are drawn upon here, and relevant examples from the course concept presented.

**Conclusion: **The design of the course corresponds to the recommendations of the GMA committee on Interprofessional Education in the Health Professions. Based on these, and considering the satisfactory evaluations, both continuation and further development of this interprofessional teaching format are justified.

## Zusammenfassung

**Einleitung:** Interprofessionelle Zusammenarbeit ist insbesondere im Bereich der klinischen Notfallmedizin von großer Bedeutung. Die an der Versorgung beteiligten Professionen müssen Hand und Hand zusammenarbeiten und Arbeitsabläufe gut koordiniert sein. Studierende der Humanmedizin und Auszubildende der Gesundheits- und Krankenpflege erhalten jedoch kaum Gelegenheit die interprofessionelle Zusammenarbeit in der Ausbildungsphase praktisch zu trainieren. Vor diesem Hintergrund haben die Klinik für Anästhesiologie und die Berufliche Schule an der Universitätsmedizin Greifswald ein Projekt zur „Erhöhung der Patientensicherheit durch die Integration von interprofessionellem Human-Factor-Training in die Ausbildung von Gesundheitsberufen“ initiiert. In dieser Darstellung soll der Frage nachgegangen werden, auf welche Weise ein interprofessionell ausgerichtetes Kursmodul der klinischen Notfallmedizin unter Maßgabe einer hohen Kompetenz- und Problemorientierung didaktisch gestaltet werden kann.

**Projektbeschreibung: **Ziel des im Zeitraum von Oktober 2013 bis September 2015 durchgeführten Projektes war die didaktische Entwicklung, praktische Etablierung und Evaluation eines simulationsbasierten und interprofessionell ausgerichteten Kursmoduls im Bereich der klinischen Notfallmedizin. Zielgruppe bildeten Studierende der Humanmedizin und Auszubildende der Gesundheits- und Krankenpflege. In der didaktischen Konzeption und Durchführung wurden moderne didaktische Modelle und Methoden genutzt. Das Projekt wurde in den Phasen der Definition, Planung, praktischen Etablierung, Evaluation und Dokumentation durchgeführt. Begleitet wurde der Projektprozess von einer systematischen Qualitätsentwicklung. In der Qualitätsentwicklung, -sicherung und Evaluation kam der Leitfaden zur Qualitätszentrierten Schulentwicklung zum Einsatz.

**Ergebnisse:** Im Zeitraum von zwei Jahren wurde ein simulationsbasiertes Kursmodul entwickelt, in den Sommersemestern 2014 und 2015 durchgeführt und evaluiert. 120 Studierende der Humanmedizin und 120 Auszubildende der Gesundheits- und Krankenpflege durchliefen das 16-stündige Lernangebot. 18 Lehrende aus Medizin und Pflege wurden zu Instruktoren/innen ausgebildet und in der Kursdurchführung von 12 studentischen Tutoren/innen unterstützt. Die zyklisch angelegten Evaluationen fokussierten auf unterschiedliche Aspekte der Projektdurchführung. Die sehr gute Benotung des Kursangebotes durch Studierende und Auszubildende weist auf eine hohe Zufriedenheit beider Berufsgruppen mit der didaktischen Gestaltung und der Qualität der Betreuung hin.

**Diskussion: **Der Ausschuss „Interprofessionelle Ausbildung in den Gesundheitsberufen“ der Gesellschaft für Medizinische Ausbildung (GMA) formuliert in einem Positionspapier Empfehlungen für die interprofessionelle Ausbildung. Die hier gegebenen Empfehlungen zur didaktischen Konzeption und Qualitätssicherung werden aufgegriffen und Entsprechungen mit dem konzipierten Kurskonzept aufgezeigt.

**Schussfolgerung: **Das entwickelte Kurskonzept entspricht im Wesentlichen den zur didaktischen Gestaltung und Qualitätsentwicklung gegebenen Empfehlungen des GMA Ausschusses „Interprofessionelle Ausbildung in den Gesundheitsberufen“. Auf dieser Grundlage und in Anbetracht der sehr guten Benotung rechtfertigt sich die Fortsetzung und Weiterentwicklung dieses interprofessionellen Lehrformates.

## 1. Introduction

Mastering clinical emergencies requires not just a high level of medical expertise, but particular skills in goal-oriented communication and effective teamwork. The professions involved in treating emergency patients must work hand in hand. Tasks must be skillfully coordinated and carried out effectively. Productive collaboration does not happen of its own accord; both intensive preparation and training are necessary [6]. Traditional and mono-professional educational structures offer little opportunity for future doctors, nurses, and other health professionals to learn in shared environments, where experience with collaborative work can be gathered. In response to the current situation, the Department of Anesthesiology and the Vocational School of Greifswald University Medical School have initiated a project to increase patient safety by integrating interprofessional human factor training into the curriculum of both health professions. The aim of this project was the content development, practical implementation and evaluation of an interprofessionally oriented and simulation-based course module on clinical emergency medicine. The target audience was comprised of medical and nursing students of University of Greifswald Medical School. This manuscript addresses how an interprofessional course module dealing with clinical emergency medicine can be pedagogically designed with a closely defined focus on competency and problem-solving. In addition, it was deemed important to identify suitable tools for systematic quality development and assurance of the presented teaching format. The present manuscript highlights aspects of implementing this project from the perspective of the educational sciences.

According to Weinert, the term competency encompasses the cognitive abilities and skills, either at the disposal of an individual or those through which abilities and skills can be learned, to solve specific problems, as well as the associated motivational, volitional and social willingness and ability to successfully apply the solutions in various situations in a responsible manner [17]. Competencies are acquired in professional and other real-world contexts and developed life-long. Professional decision-making skills, as a precisely defined concept, refer to the concrete actions taken by a person in the course of performing their work. Professional decision making skills are to be understood as a combination of professional and social skills and self-knowledge. Methodological competency, communication and learning skills are intrinsic to these competency areas [13]. The ability to make decisions reflexively indicates an ability beyond professional decision making skills to also grasp organizational conditions for working and learning, and to critically reflect upon them. The ability to assume a more distanced view of a concrete work situation also enables the individual to critically question how tasks and processes are organized within the context of existing occupational and social structures, and to understand them in relation to oneized within the context of exis [1]. The definitions of competency elucidated above provide the basis for the description and discussion to follow.

## 2. Project description

### 2.1. Project aims

**Figure 1:**
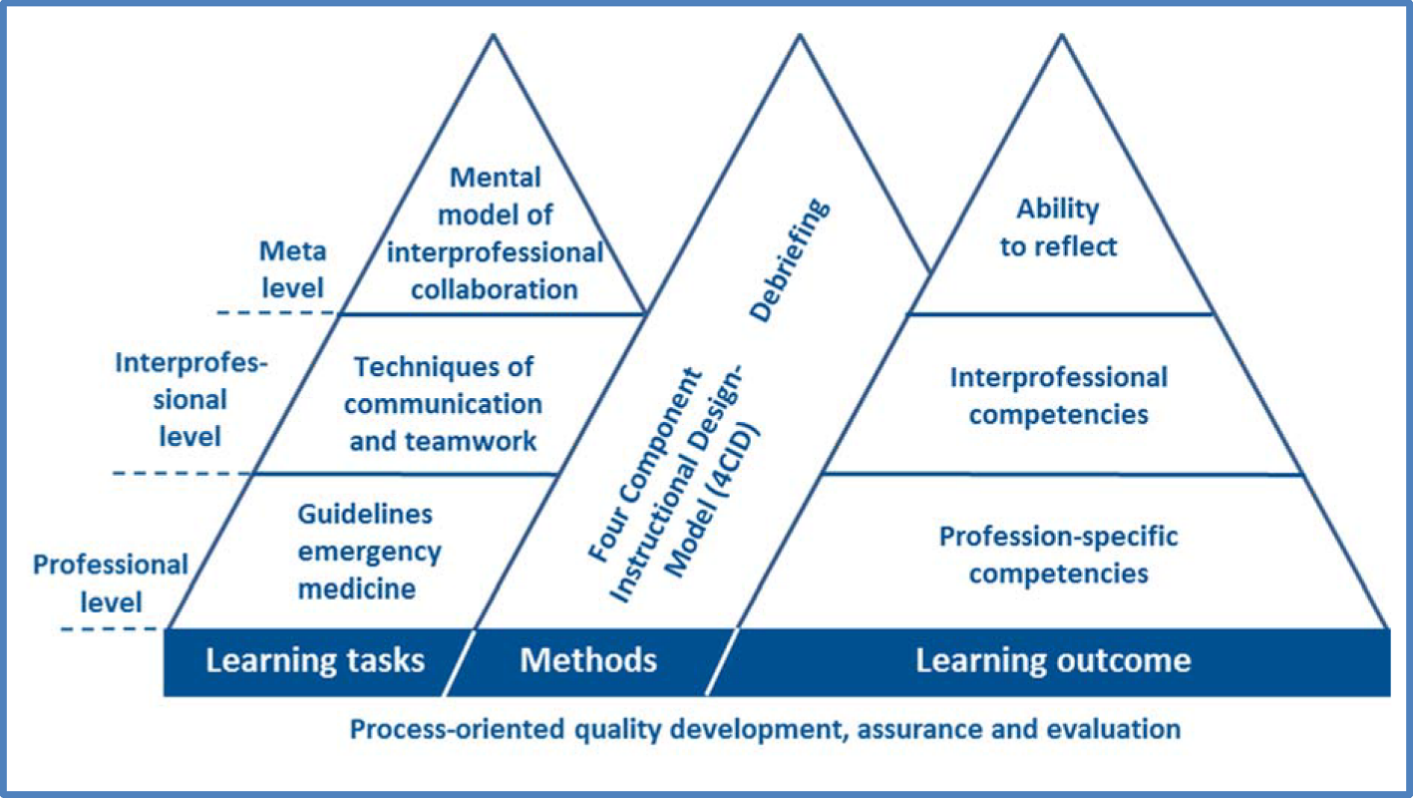
Design of the course module on interprofessional learning in clinical emergency medicine

The project goal was to develop course content, to implement and to evaluate a simulation-based course module in clinical emergency medicine to be jointly attended by both, university and vocational students. The design and teaching of a four-day training session for instructors of both professions and student tutors was an integral component of the project, although not the focus of this manuscript. The team entrusted with conducting the project consisted of anesthesiologists, clinical nursing instructors, vocational school instructors, and an educational specialist who was also in charge of project coordination. The process went through the phases of definition, planning, practical implementation, evaluation and documentation. Points of intersection between the curricular requirements and commonalities in the conditions for participation were first identified in project team meetings. Based on these, course content and learning objectives were defined and course design and implementation were planned, along with identification of measures for quality development and assurance, and evaluation (see figure 1 (Fig. 1)).

#### 2.2. Pedagogical design of the course module

##### 2.2.1. Learning objectives

The stated learning objective was to impart relevant skills in making professional decisions to the students from both professions in accordance with their learning and educational needs. In detail, it was intended to have participants find themselves in a position to examine an emergency patient in a structured manner, recognize life-threatening conditions, and take required and appropriate action. This also encompassed aspects that give healthcare professionals the ability to apply their knowledge of effective team actions, often under the difficult and busy circumstances that accompany a medical emergency [7]. The aspect of interprofessional work explicitly mentioned here requires, in particular, the ability to collaborate and communicate clearly and effectively in a team. As a result, the defined learning objectives reflect both professional and interprofessional levels. The professional level involves knowledge of established routines and responses in emergency medicine, whereas the interprofessional level involves the use of communication techniques and working in groups. While the professional level leads to the acquisition of occupation-specific decision-making skills, the interprofessional one deals with a dimension of professional decision-making that spans across more than one profession. From an educational standpoint, the collaborative creation of a shared mental model for interprofessional cooperation was intended, with learning reflected action as a goal.

##### 2.2.2. Curricular content

Two established mnemonics describing medical responses to emergencies formed the basis for designing the subject-related content of this course module. The ABCDE approach (A=airway, B=breathing, C=circulation, D=disability, E=environment) is a mnemonic device that enables a methodical approach to assessing emergency medical situations [13]. The goal in using this device is to prioritize diagnostics and therapy so that life-threatening symptoms in a patient are treated first. The SAMPLE history (S=symptoms, A=allergies, M=medications, P=personal health history, L=last meal, E=environment/events) is used primarily in pre-clinical emergency care serving to ensure methodical anamnesis and effective communication of information about the patient to those directly involved in providing further care.

Serving as the reference work for the interprofessional design was the Crew Resource Management (CRM) approach. CRM specifically sharpens the awareness that the type and manner of communication and the nature of the relationships between the team members are crucial to rational and focused action in critical situations. The overarching aim of CRM is not only to reduce the rate of complications and incidences (preventive approach), but also to be able to act more effectively and accurately when managing incidences (reactive approach) [7]. Fifteen recommendations are made regarding focusing attention, providing leadership, making decisions, team coordination and communication; these are presented to the students in the form of concrete techniques [7].

##### 2.2.3. Pedagogical models and methods

**Figure 2:**
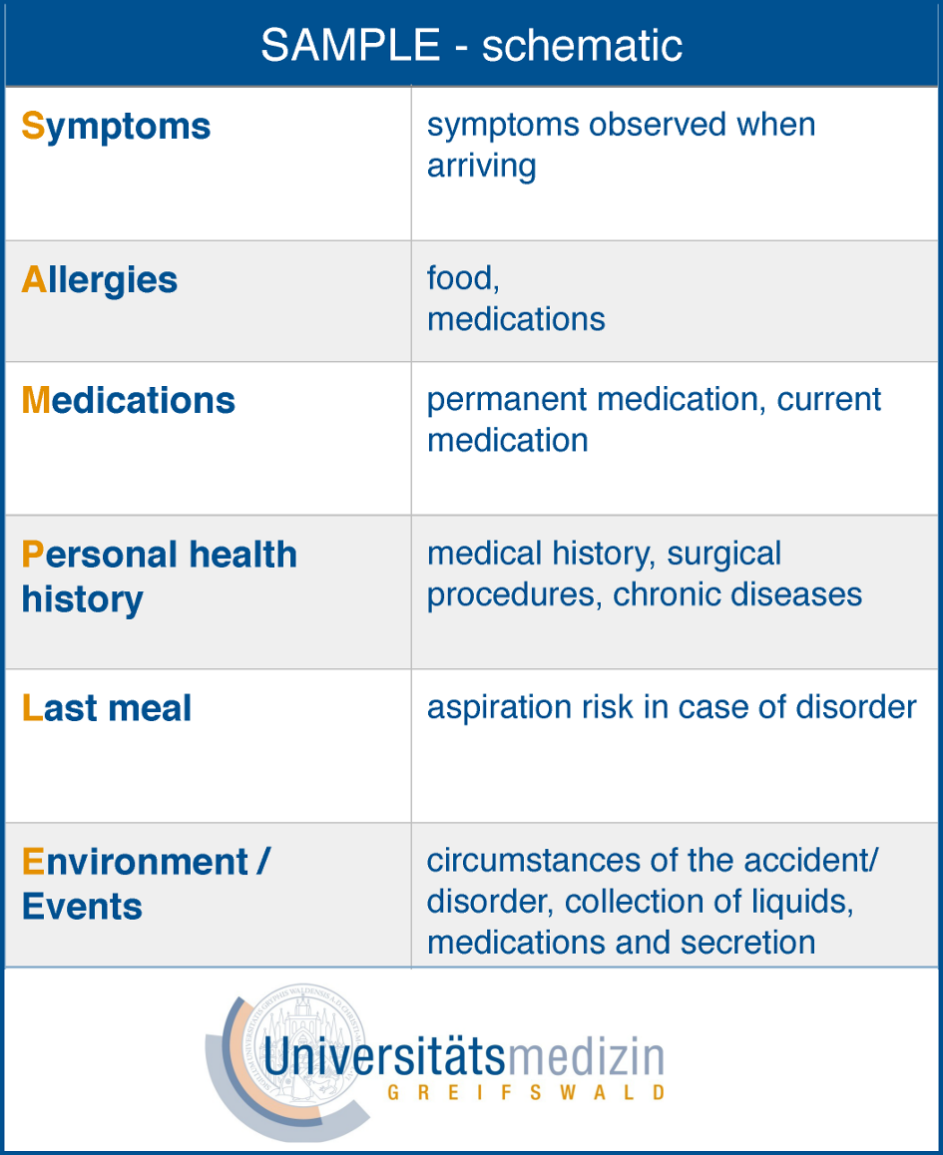
SAMPLE flashcard

The four-component instructional design model (referred to as 4CID) by Van Merriënboer was drawn upon to develop course content on the professional and interprofessional levels [14]. 4CID is an empirically investigated model [10] based on theoretical assumptions about course design and complex learning. It also complies with the recommendations for situated learning [3]. The 4CID-model recommends drafting both, authentic assignments and materials for teaching and learning that assist the learning process. The starting point for designing the learning sessions is to inquire about what students need to know and be able to do in order to demonstrate complex cognitive abilities in comprehensive and concrete terms. In this case, they eventually need to provide emergency care to a patient as a team. In a systematic process involving ten steps, eight emergency case scenarios, three skill stations (cardiopulmonary resuscitation, airway management, intravenous access), and supporting teaching materials (lectures) and learning materials (flashcards [see figure 2 (Fig. 2)]) were developed.

An essential part of the pedagogical design was the follow-up discussions of the case scenarios in the form of a debriefing session [2]. By applying this method, it was possible to work specifically towards the intended goal of acting in a reflected manner, in addition to acquiring skills in professional decision-making.

##### 2.2.4. Creating the learning environment

The case scenarios developed according to 4CID were integrated into a simulated learning environment and designed following a high-fidelity approach [9], [15]. High-tech mannequins (life-sized dolls) were used to simulate a number of vital parameters and respond to treatments, such as the administration of oxygen, medication etc. Medical equipment, supplies and, to the extent possible, drugs were provided in their original formulation. The learning environment was set up to resemble a hospital room. The degree to which real conditions were created increased the probability of knowledge transfer to similar situational contexts [9].

#### 2.3. Quality assurance

An established method of quality-centered school development was applied to the quality development of the project [8]. Working materials involving quality areas, guidelines, criteria, indicators and standards were developed and provided in a manual. The quality standards were assessed by regular, formative evaluations, and the course underwent continual development based on evaluation results.

#### 2.4. Practical implementation

**Figure 3:**
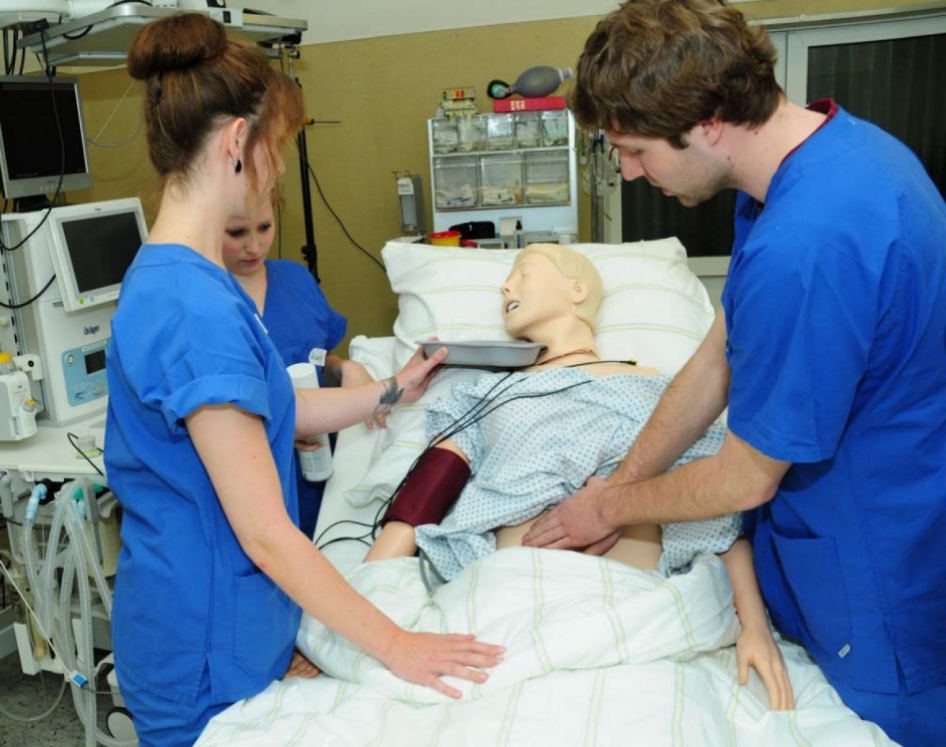
Medical and vocational students practice cooperation in medical emergencies

Two-day course modules were attended six medical students and vocational students, respectively. The courses were taught by an interprofessional team of four instructors and two student tutors. Following an introduction to the theory behind the mnemonics and techniques, and after practicing the individual skills at the skill stations, course participants were asked to practically apply their knowledge in simulated case scenarios. The assignment was to examine a simulated patient in a hospital setting as an interprofessional team, to make a working diagnosis, and to undertake necessary actions as a team (see figure 3 (Fig. 3)). To encourage problem-solving, the use of learning materials, such as flashcards, was permitted. From the beginning of the course, it was important to manage complex emergency situations. The level of difficulty increased as the course progressed. In dynamic medical situations, where critical decisions about patient care must be made quickly, the team members were required to repeatedly organize themselves, verbalize the actions to be taken and strategies to be followed, assign tasks to team members, and prioritize medical measures in order to move forward toward solving the problem. Arising questions included:

- Who assumes the role of team leader?
- Are orders clearly worded?
- In what manner do responses or feedback take place?.

Debriefing sessions took place directly after each case scenario, during which the team members were asked by the instructors to share their personal impressions and reactions, and to jointly reconstruct how the scenario unfolded. Subjective experiences of success and difficulty in the process of providing medical treatment, in communicating and working as a team were discussed and analyzed. Reflection was encouraged through open-ended questions posed by the instructors, and alternative actions and options for conducting oneself were discussed together. The debriefing closed with a take-home message in which the participants consolidated important aspects of their learning process in succinct statements.

## 3. Results

### 3.1. Effects of the educational design

In the safe learning context of a simulated environment, participants were asked to appropriately apply the acquired techniques and approaches in different situations. Participants sustainably expanded on their prior knowledge, the learned approaches for emergency medical response, communication techniques, and teamwork, by engaging in concrete action. By working together, they identified occasions where their spheres of work intersect, recognized differences in forms of knowledge and responsibilities, as well as the relevance of effective communication and teamwork.

Cooperation did not always happen smoothly. If working diagnoses were not clearly communicated within the team, medical treatment could usually not be carried out consistently. Knowledge regarding the responsibilities and competencies of the various team members involved in providing the medical care was also insufficient. Problems that hindered effective and targeted care of the simulated patients arose during collaboration.

The significance of the debriefing sessions became apparent as an essential part of the didactic approach. When reconstructing the scenarios, team members were encouraged to articulate their subjective assessments of the treatment process and the cooperation of the team. Taking the role of moderator, instructors emphasized the diverse, profession-specific viewpoints in a particular situation, thereby enabling the team members to clarify misunderstandings and solve conflicts. The acquired knowledge and insights were then practiced in the form of concrete behavioral changes in the subsequent case scenarios. In this manner, the participants learned professional decision-making skills on a profession-specific and interprofessional level. In addition, open-ended questions asked by the instructors unveiled stereotypes of team member roles as the most frequent cause of individual cognitive conflicts [11]. This critical engagement with one's own and others' professional identities in the context of profession-specific spheres of responsibility and cirumstantial factors positively influenced the development of the ability to take reflected action.

#### 3.2. Project results

Over the course of the project, a simulation-based course was designed according to modern and theoretically confirmed teaching models and methods. Following the procedures of 4CID, eight case scenarios, three skill stations and the accompanying teaching and learning materials were developed. In the 2014 and 2015 summer semesters, 20 course modules were offered and attended by 120 medical students and 120 nursing students. The design and conduction of a four-day instructor training session was an integral component of this project. Eighteen physicians, nursing instructors, nurses, and nursing school instructors, as well as 12 students were trained as course instructors and tutors, respectively. As of the 2014 summer semester, the course module has been permanently integrated into the curriculum for the second year of nursing education at the nursing schools. Inclusion into the curriculum for medical students is planned for the beginning of the 2016 summer semester. With the presentation of this project at conferences and workshops, the experience gathered in designing and teaching an interprofessional course has been made available to interested audiences. Upon project completion, an interdisciplinary working group was formed to develop an overall interprofessional learning concept across multiple institutions for at the University of Greifswald Medical School.

#### 3.3. Results of the quality assurance measures

An established quality-centered school development tool proved to be a suitable instrument for quality development and evaluation of the course module. Methods for empirical social and education research, which focused on different aspects of the project, were used in the evaluation.

Student satisfaction with the course module was documented using a standardized questionnaire covering aspects of the pedagogical design and quality of teaching. On a scale from 1 to 5 (1=excellent, 5= inadequate), initial mean ratings were 1.34 for medical students (n=96; SD=0.52), and 1.37 for nursing students (n=102; SD=0.49). This evaluation indicates a high level of satisfaction with the course design and the quality of teaching.

The case scenarios were recorded on video as a form of open non-participatory observation [4]. By using an analytical software tool, it was possible to quantitatively and reproducibly record and analyse observable events. Thereby, assertions could be made about the extent to which defined learning objectives had been achieved on a profession-specific and interprofessional level. The summative evaluation results have not yet become available at the time of this publication.

Following an explorative approach, interviews were conducted with students from both professional groups. When analyzing the data, the questions concerning the extent to which the participants reflected on their roles and attitudes, and which experiences or structural elements of the course exerted influence on these reflections, were to be pursued. The results of the data analysis will be scientifically evaluated in two diploma-theses in the field of Psychology at the University of Greifswald.

## 4. Discussion

Over two years, a simulation-based course module in clinical emergency medicine involving interprofessional learning was developed, implemented and evaluated. Appropriate tools for quality development and assurance were identified and applied. The course evaluations indicate that this concept is suitable for interprofessional education in the health professions.

In light of the activity reported within this manuscript, the recommendations made in the position paper of the GMA committee on Interprofessional Education in the Health Professions are suitable in particular in the areas of curriculum design and teaching concepts" and "quality assurance and evaluation [16].

Over the course of the project, a theoretically established course concept was developed, the practical implementation of which required the prior training of instructors. The pedagogical approach involved a strong emphasis on competency, problem-solving, decision-making, and specific emergency situations. Reflective phases were a structural component of practical, case-based learning in a joint curricular activity. An interprofessional project team identified overlapping learning content, and a course concept was then developed in cooperation with the affected professional groups. Topics relevant to all groups, such as communication and teamwork, were an essential part of the curricular design. 

The recommendations for quality assurance and evaluation were complied with to the extent that the defined learning objectives were designed according to an established educational theory of competency. Using a systematic procedure in alignment with quality-centered school development, learning objectives were formulated, documented (survey, observation), and evaluated.

The excellent ratings reflect the high level of satisfaction with the concept of the course module and the quality of teaching on the part of students. An equally high level of acceptance was seen in both student groups in regard to the educational format.

The compliance of the applied learning strategy with the requirements of the German National Competency-based Catalogue of Learning Objectives for Undergraduate Medical Study [http://www.nklm.de] could not be assessed, since these were not yet available at the time of project planning and implementation.

## 5. Conclusion

It has been recognized that interprofessional teaching formats must be developed for the health professions to further improve medical care and patient safety, particularly in the area of clinical emergency medicine. Based on its adherence to the recommendations given by the GMA committee on Interprofessional Education in the Health Professions, and the excellent student evaluations of the course module presented here, the teaching and learning format developed for the course module has proven to be suitable and, as such, will be continued and developed in the future.

## Funding

As part of the program “Operation Team: Interprofessional Learning in the Health Care Professions”, this project received financial support from the Robert Bosch Foundation and the project interStudies (number 01PL12039) from the University Greifswald. 

## Acknowledgements

We wish to thank Sportstec Germany GmbH in Pöcking for the provision of video analysis software, MTO Psychologische Forschung und Beratung GmbH in Tübingen for providing the guidelines on quality-centered school development available, and the InPASS Institut für Patientensicherheit und Teamtraining GmbH in Reutlingen for supplying learning materials. We also extend our thanks to Dr. Martin von der Heyden and Erik Eichhorn for planning and conducting the instructor training sessions, and all instructors who took part in the project, especially Anja Tessler, Sandra Huber, Annika Nowack, Sandra Wodrig, Änne Otto, Carolina Hornke, Thomas Ratay, Kai Sommer, Eik Schäfer, Steffen Dickel, and Patrick Adler.

## Competing interests

The authors declare that they have no competing interests.
